# Coronary recanalization following spring coil occlusion: A case report and literature review

**DOI:** 10.1097/MD.0000000000041929

**Published:** 2025-03-14

**Authors:** Chuanmin Jian, Song Liu, Kang Song, Jie Zhou, Shaoting Shi

**Affiliations:** a Department of Cardiovascular Medicine, The Affiliated Hospital of Qingdao University, Qingdao, China; b Department of Cardiovascular Medicine, Qingdao University, Qingdao, China; c Department of Cardiovascular Medicine, Qingdao Municipal Hospital, Qingdao, China; d Department of Radiation Oncology, The Affiliated Hospital of Qingdao University, Qingdao, China.

**Keywords:** case report, coronary arteriovenous fistula, coronary artery perforation, coronary heart disease intervention, spring coil occlusion

## Abstract

**Rationale:**

Coronary artery perforation (CAP) is one of the most serious complications of percutaneous coronary intervention. Coronary arteriovenous fistula is a considerably rare type of congenital CAP. They are usually difficult to distinguish.

**Patient concerns:**

A male patient developed coronary artery perforation during percutaneous coronary intervention. As balloon occlusion was ineffective, a decision was made to implant a spring coil and bypass the occluded segment. However, the placement of spring coils restored patency in the occluded distal segment of the right coronary artery (RCA).

**Diagnoses:**

Coronary computed tomography angiography is an auxiliary tool and Digital Subtraction Angiography(DSA) is the gold standard for the diagnosis.

**Interventions:**

Surgery and implant a spring coil are the main treatment methods.

**Outcomes:**

The patient’s RCA regained its blood supply. Coronary artery recanalization arteries occurred after spring coil placement. Combined with the imaging presentation, we finally determined that he was coronary-right ventricular fistula.

**Lessons:**

Although congenital CAPs are rare, appropriate detection and timely confirmation by coronary angiography are important for determining their subsequent management. Congenital coronary arteriovenous fistulae may be considered when coronary artery perforation during percutaneous coronary intervention, balloon blockade is ineffective and the patient’s vital signs are stable.

## 1. Introduction

Coronary artery perforation (CAP) is one of the most serious complications of percutaneous coronary intervention. The management of CAP usually involves prolonged balloon inflation, reversal of anticoagulation, and covered stent implantation.^[1,2]^ In some cases, a spring coil is used to seal the perforation site and bypass the affected segment of the vessel; this prevents prolonged bleeding, which may lead to pericardial tamponade.^[3]^ In this context, a coronary arteriovenous fistula (CAF) is a considerably rare type of congenital CAP.^[4]^ CAFs are usually found in the right coronary artery (RCA), and they direct the flow into the right cardiac system. Here, we describe a case of a CAF which was initially considered to be an artificially induced coronary perforation, and treated accordingly. This led to recanalization and smooth blood flow in the occluded segment of the affected coronary artery.

## 2. Case description

**Figure 1.**
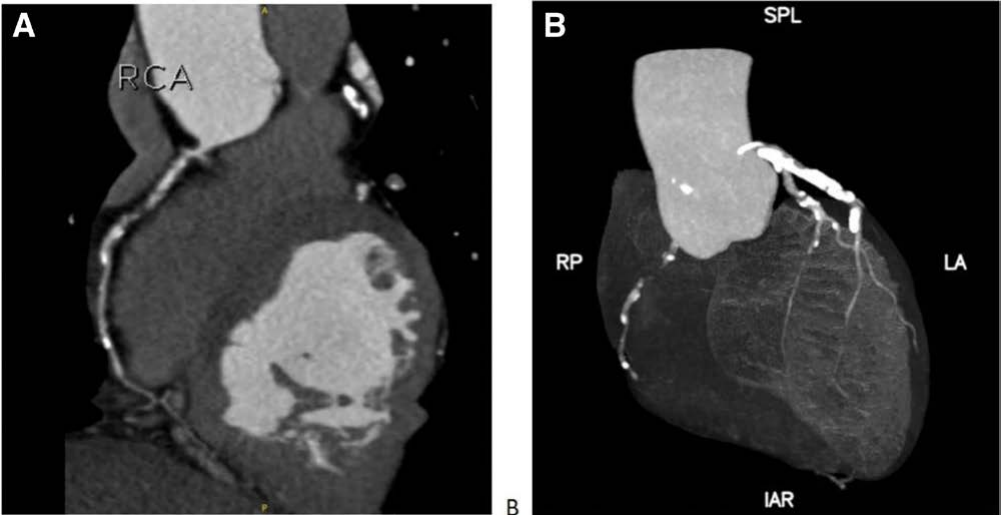
Findings on preoperative coronary computed tomography angiography. IAR = inferior anterior right, LA = left anterior, RCA = right coronary artery, RP = right posterior, SPL = superior posterior lateral.

A 67-year-old male patient presented to our hospital on March 13, 2024 with a history of chest discomfort, which had lasted for 5 years and had aggravated for the past 1 month. The patient had first experienced poorly localized right-sided chest discomfort 5 years previously, following physical activity. However, he did not seek treatment as he was able to engage in light-to-moderate physical activity and the attacks were infrequent. He had attended the outpatient clinic of the cardiovascular medicine department 1 month previously owing to frequent chest pain during activities. Coronary computed tomography angiography, which was performed on March 11, 2024, showed calcification of the RCA with severe localized stenosis in the proximal part of the lumen. The left coronary artery also demonstrated calcification with mild to moderate luminal narrowing (Fig. 1). The attending outpatient physician diagnosed him with unstable angina and coronary atherosclerotic disease and admitted him to the hospital. Notably, the patient was previously healthy and showed no remarkable signs on physical examination at admission.

The diagnosis was confirmed during hospitalization, and treatment was initiated with dual antiplatelet therapy, lipid regulating and anticoagulant drugs, and coronary vasodilators. On preoperative evaluation, the levels of the following parameters were found to be out-of-range: cardiac troponin (0.054 μg/L), low-density lipoprotein-cholesterol (3.61 mmol/L), high-density lipoprotein-cholesterol (1.61 mmol/L), apolipoprotein B (1.14 g/L), total cholesterol (5.88 mmol/L), and lipoprotein (a) (1756 mg/L); the other laboratory indicators were within the normal ranges. Electrocardiography demonstrated sinus rhythm with a nonspecific T-wave abnormality, and cardiac color Doppler examination showed post-myocardial infarction changes, segmental motion abnormalities of the left ventricular wall, and an ejection fraction of 53%.

**Figure 2.**
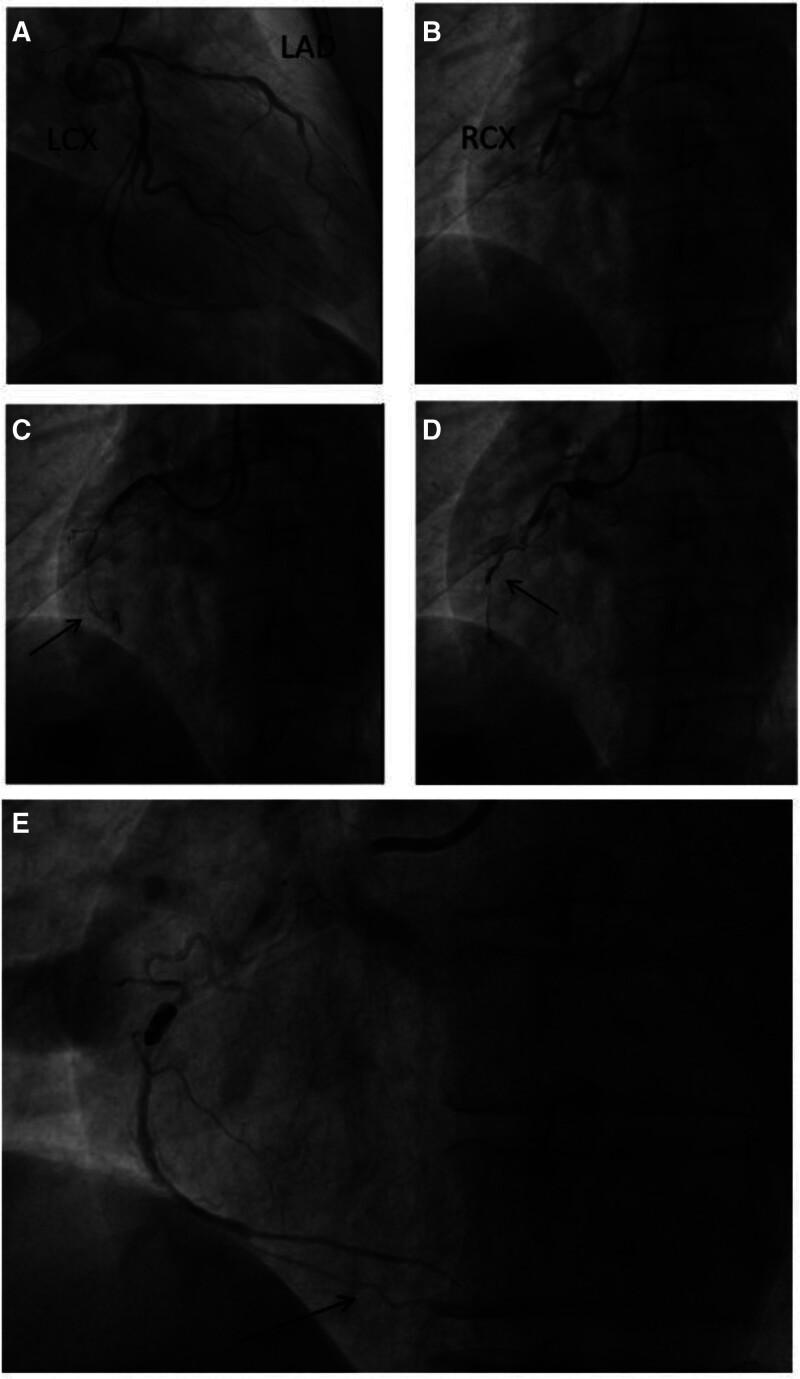
Findings on preoperative digital subtraction angiography. (A) Left coronary artery. (B) Right coronary artery. (C) Site of rapid dissipation of contrast medium. (D) Spring coil blocking position. (E) Distal right coronary artery. LAD = left anterior descending, LCX = left circumflex, RCX = right coronary artery.

Coronary angiography was performed under cardiac monitoring on March 14, 2024 at the Interventional Center, after excluding any contraindications. The findings showed the coronary distribution to be left dominant. The caudal segment of the left main coronary artery demonstrated 30% stenosis with calcification, and a diffuse calcified lesion was observed in the left anterior descending coronary artery with 85% stenosis at the narrowest point. The middle segment of the circumflex artery showed 70% stenosis with calcification and the proximal RCA demonstrated 60% stenosis with occlusion of the middle segment (Fig. 2A, B). Although percutaneous coronary intervention was attempted in the RCA, the distal segment of this artery could not be visualized. A pilot wire was therefore introduced into the distal segment, and the completely occluded mid-section was predilated using a 1.5 × 15 mm Maverick balloon (10 atm/5 seconds). During the dilatation process, digital subtraction angiography showed mid-vessel localization and rapid extravasation of the contrast agent (Fig. 2C). This was considered to be indicative of coronary artery perforation, and the proximal end of the leak was blocked with a 1.5 × 15 mm Maverick balloon (4 atm). Bedside color Doppler echocardiography did not show any evidence of contrast agent leakage into the pericardial cavity. Digital subtraction angiography performed 30 minutes after intermittent blockade showed persistent contrast agent extravasation; however, no significant pericardial effusion was observed on cardiac color Doppler examination. On urgent consultation, the Department of Interventional Medicine provided 2 releasable spring coils (Target 360 Soft 2 mm × 4 cm [M0035472040] and Target 360 Ultra 2 mm × 6 cm [M0035422060]; Stryker Inc.) for blocking the middle segment of the perforated RCA (Fig. 2D). The leakage disappeared after placement of the coils, and the distal segment of the blocked posterior RCA was seen to have a patent lumen (Fig. 2E). Repeat cardiac color Doppler examination demonstrated no evidence of significant pericardial effusion; the procedure was therefore concluded and the patient was sent to the ward. During the postoperative period, the patient did not experience any significant discomfort and had fewer symptoms than before. Our team intended to wait for a brief period to allow the patient to recover before managing the lesion in the left coronary artery. Patients and their families actively engaged in the comprehensive treatment process and consented to the prescribed treatment modalities. Historical and current information from this episode of care organized as a timeline (Table 1).

**Table 1:** Timeline.

5 yr ago
The patient had first experienced poorly localized right-sided chest discomfort 5 yr previously, following physical activity. However, he did not seek treatment as he was able to engage in light-to-moderate physical activity and the attacks were infrequent
1 mo ago
He had attended the outpatient clinic of the cardiovascular medicine department 1 mo previously owing to frequent chest pain during activities
Outpatient visits
Coronary computed tomography angiography showed calcification of the coronary artery with stenosis
Intraoperative
Digital subtraction angiography showed mid-vessel localization and rapid extravasation of the contrast agent
30 min later
The use of balloon occlusion was found to be ineffective, as indicated by echocardiography showing no evidence of pericardial effusion. It is recommended to proceed with spring ring blocking
1 h later
The leakage disappeared after placement of the coils
1 d later
Repeat cardiac color Doppler examination demonstrated no evidence of significant pericardial effusion
3 d later
The patient was discharged

## 3. Discussion

This report describes a case of chronic total coronary occlusion, as demonstrated by coronary angiography. Intraoperative contrast artery extravasation was observed during balloon dilatation of the stenosed RCA. Digital subtraction angiography showed rapid dissipation of the contrast agent despite balloon blockade, which lasted for approximately 30 minutes. No pericardial effusion was observed during or after the procedure. This suggested that the contrast agent did not enter the pericardial cavity, and flowed into the ventricle instead; the blood also flowed into the circulation via the ventricle. After successful blockade of the extravasation site (at the mid-RCA) with a spring coil, the previously occluded distal segment of the artery was visualized; the lumen was patent and demonstrated increased blood flow.

Vascular dissection and perforation during balloon dilatation and stenting of coronary arteries is uncommon, and vascular dissection and perforation most often occurs in vessels with chronic total occlusion, severe calcification, or tortuous tortuosity during procedures.^[1]^ As the coronary arteries are positioned on the surface of the heart, blood may enter the pericardial cavity if they are ruptured. Some patients with ruptured coronary arteries may therefore develop pericardial tamponade or even cardiac arrest due to pericardial effusion.^[5]^ Our patient did not develop pericardial effusion, and the pattern of rapid extravasation of contrast during coronary angiography was suggestive of leakage into the ventricular cavity. It was initially believed that the patient had a history of an occult myocardial infarction which had led to thinning of the ventricular wall, and a sharp thrust from the guidewire (as it passed through the occluded segment) had ruptured the vessel wall causing extravasation into the ventricular cavity. However, the extravasation continued despite balloon occlusion, and the patient did not experience any discomfort during occlusion, which lasted for 30 minutes. In addition, the distal segment of the RCA was visualized after successful occlusion using a spring coil, and the blood flow was observed to be smooth. The patient was therefore considered to have a coronary-right ventricular fistula, which had previously caused the blood to flow from the middle segment of the RCA to the right ventricle; the coronary artery proximal to the fistula was subsequently occluded by an atherosclerotic plaque. Balloon dilatation restored patency of the vessel proximal to the fistula and reinitiated blood flow through the latter (to the right ventricle). As the fistula was a congenital lesion, balloon dilatation failed to seal the leak (unlike iatrogenic coronary artery perforation). However, spring coil placement for sealing the middle segment of the RCA successfully sealed the fistula. It also allowed greater blood flow into the already patent vessel; this allowed visualization of the distal trunk of the RCA. Secure fastening of the spring coil prevented it from being dislodged and displaced into the ventricle.

CAP is one of the most serious complications of percutaneous coronary intervention. The classical types of CAP were proposed by Ellis in 1994; these are as follows: type I: contrast fills the lumen to form a localized niche without significant extravasation, type II: contrast extravasates into the pericardium or myocardium via an orifice measuring <1 mm, and type III: contrast extravasates via an orifice measuring >1 mm and extravasation or hemorrhage occurs into another vascular lumen.^[6]^ The usual management of CAP involves prolonged balloon inflation, reversal of anticoagulation, and covered stent implantation.^[1,2]^ Persistent intraoperative balloon occlusion or overlay stenting is occasionally unsuccessful. Coronary artery bypass grafting or emergency surgical repair is needed in these cases to avoid adverse cardiac events (such as pericardial tamponade, myocardial injury, or even myocardial infarction) consequent to prolonged ischemia; few patients also require vascular embolization.^[3]^

Vascular embolization therapy is usually employed in cases of hemorrhage, aneurysms, arteriovenous malformations, and tumors. Mechanical occlusion devices (coils, stents, and balloons, among others), autologous materials (blood clots or subcutaneous fat particles, among others), particulate materials (polyvinyl alcohol particulates, gelatin embolics, trisacryl gelatin microspheres, and chitosan, among others), and liquids/gels (ethanol and calcium alginate, among others) are usually used.^[7]^ Spring coils are commonly used to completely block blood flow to the diseased vessel. In some organs with a rich collateral circulation (such as the brain and kidneys), blocking a certain segment has less impact on the blood supply to normal tissues. However, the myocardium is usually supplied by a single vessel; occlusion of any 1 vessel may therefore cause localized cardiomyocyte death owing to ischemia, and thereby cause or aggravate myocardial infarction. In this context, spring coils are commonly used to treat congenital heart disease (such as patent ductus arteriosus), CAFs, coronary artery aneurysms, and arterial side branch steal syndrome after coronary artery bypass grafting.^[8]^ Extreme caution needs to be exercised when using these coils to occlude coronary arteries. They may only be used to occlude smaller vessels with a small distribution area or vascular segments with a rich collateral circulation (to prevent jeopardizing myocardial blood supply). Coil placement (to occlude and bypass a ruptured segment of a vessel) is needed to address persistent extravasation which leads to pericardial tamponade, as this can have life-threatening consequences.

In this context, patients with CAP demonstrate a high incidence of adverse events, which commonly include in-hospital death.^[9]^ These patients have 5-fold higher 30-day postoperative mortality rates than those without CAP.^[10]^ It is therefore essential that ruptured coronary arteries are treated promptly. CAFs are a type of congenital CAP; they have a considerably low incidence rate and an estimated prevalence rate of 0.2% in the general population.^[4]^ These fistulas mostly occur independently (80%), but may occur in association with other cardiac malformations (20%) such as atrial or ventricular septal defects, tetralogy of Fallot, and arterial ductus arteriosus.^[11]^ CAFs usually occur in the RCA, and mostly allow flow into the right cardiac system; however, they do allow flow into the pulmonary artery and left cardiac system to a lesser extent. In some cases, these fistulas may be occluded by lesions such as coronary atherosclerotic plaques or thrombi; they may also be caused by coronary artery atherosclerosis, arteritis, and trauma.^[11,12]^

The clinical symptoms of CAFs mainly depend on their location (as in a large coronary vessel) and the volume of fractional flow. Children are more likely to be symptomatic, and many asymptomatic patients usually demonstrate ischemia only on electrocardiography. Therapeutic measures in cases of symptomatic CAF include surgical and transcatheter interventions; in particular, spring coil occlusion has demonstrated significant clinical efficacy. Our patient had a dominant left coronary artery with occlusion of the mid-section of the RCA. As balloon occlusion was ineffective after contrast extravasation, a spring coil was used to occlude the vessel and bypass this segment. Surprisingly, the balloon restored patency of the distal segment of the RCA instead of occluding it; this indicated that the rupture was not iatrogenic. In addition, contrast extravasation was observed at a site where CAFs are frequently found. In view of these findings, it was determined that the contrast extravasation in this patient was attributed to his CAF.

## 4. Conclusion

In conclusion, this report describes a rare case of coronary artery recanalization after spring coil placement. Numerous findings indicated the presence of a CAF in this case. Although congenital CAPs are rare, appropriate detection and timely confirmation by coronary angiography are important for determining their subsequent management.

## Author contributions

**Investigation:** Chuanmin Jian, Song Liu, Shaoting Shi.

**Methodology:** Song Liu.

**Writing – original draft:** Chuanmin Jian, Song Liu, Kang Song.

**Writing – review & editing:** Chuanmin Jian, Jie Zhou.

## References

[1] Avula V Karacsonyi J Kostantinis S. Incidence, treatment, and outcomes of coronary artery perforation during percutaneous coronary intervention. J Invasive Cardiol. 2022;34:E499–504. 35714223 10.25270/jic/21.00358

[2] Abdalwahab A Farag M Brilakis ES Galassi AR Egred M. Management of coronary artery perforation. Cardiovasc Revasc Med. 2021;26:55–60. 33203580 10.1016/j.carrev.2020.11.013

[3] Al-Lamee R Ielasi A Latib A. Incidence, predictors, management, immediate and long-term outcomes following Grade III coronary perforation. JACC Cardiovasc Interv. 2011;4:87–95. 21251634 10.1016/j.jcin.2010.08.026

[4] Leong KE Joshi S Grigg L. Misguided diversions: coronary artery fistulae. Eur Heart J. 2017;38:2150. 28407098 10.1093/eurheartj/ehx185

[5] Lemmert ME van Bommel RJ Diletti R. Clinical characteristics and management of coronary artery perforations: a single-center 11-year experience and practical overview. J Am Heart Assoc. 2017;6:e007049. 28939719 10.1161/JAHA.117.007049 PMC5634316

[6] Ellis SG Ajluni S Arnold AZ. Increased coronary perforation in the new device era. Incidence, classification, management, and outcome. Circulation. 1994;90:2725–30. 7994814 10.1161/01.cir.90.6.2725

[7] Hu J Albadawi H Chong BW. Advances in biomaterials and technologies for vascular embolization. Adv Mater. 2019;31:e1901071. 31168915 10.1002/adma.201901071 PMC7014563

[8] Loh SX Brilakis E Gasparini G. Coils embolization use for coronary procedures: basics, indications, and techniques. Catheter Cardiovasc Interv. 2023;102:900–11. 37668102 10.1002/ccd.30821

[9] Hirai T Nicholson WJ Sapontis J.; OPEN-CTO Study Group. A detailed analysis of perforations during chronic total occlusion angioplasty. JACC Cardiovasc Interv. 2019;12:1902–12. 31255554 10.1016/j.jcin.2019.05.024

[10] Kinnaird T Anderson R Ossei-Gerning N.; British Cardiovascular Intervention Society and the National Institute for Cardiovascular Outcomes Research. Legacy effect of coronary perforation complicating percutaneous coronary intervention for chronic total occlusive disease: an analysis of 26 807 cases from the British Cardiovascular Intervention Society Database. Circ Cardiovasc Interv. 2017;10:e004642. 28500138 10.1161/CIRCINTERVENTIONS.116.004642

[11] Buccheri D Chirco PR Geraci S Caramanno G Cortese B. Coronary artery fistulae: anatomy, diagnosis and management strategies. Heart Lung Circ. 2018;27:940–51. 29503240 10.1016/j.hlc.2017.07.014

[12] Gowda RM Vasavada BC Khan IA. Coronary artery fistulas: clinical and therapeutic considerations. Int J Cardiol. 2006;107:7–10. 16125261 10.1016/j.ijcard.2005.01.067

